# The role of vein grafts in reconstructive head and neck microsurgery

**DOI:** 10.1016/j.bjorl.2021.09.004

**Published:** 2021-10-26

**Authors:** Chih-Sheng Lai, Yi-Ting Chang, Ching-Hui Shen, Yueh-Chi Tsai, Chen-Te Lu, Jung-Hsing Yen, I-Chen Chen, Yi-Ling Lin

**Affiliations:** a National Chung Hsing University, Department of Post-Baccalaureate Medicine, Taichung, Taiwan, Republic of China; b Taichung Veterans General Hospital, Department of Anesthesiology, Taichung, Taiwan, Republic of China; c National Yang Ming Chiao Tung University, Faculty of Medicine, School of Medicine, Taipei, Taiwan; d HungKuang University, Department of Nursing, Taichung, Taiwan, Republic of China; e Taichung Veterans General Hospital, Department of Emergency, Division of Trauma and Critical Care Medicine, Taichung, Taiwan, Republic of China

**Keywords:** Interposition vein graft, Arteriovenous loop, A–V loop

## Abstract

**Objective:**

Free tissue transfer is widely used for head and neck reconstruction. In certain circumstances, vein grafting is required to elongate free flap pedicles to connect them to appropriate recipient vessels. Because of controversy regarding the use of interposition vein grafts in free tissue reconstruction, this paper reports vein graft indications, techniques, safety, and outcomes for head and neck microvascular surgery.

**Methods:**

Twenty-six patients (23 men and 3 women) who underwent interposition vein grafting concurrent with free tissue transfer were included in this study. The most common reason for head and neck reconstruction with vein graft was tumor recurrence, followed by flap salvage. The interposition vein grafts were applied in two manners as temporary arteriovenous (A–V) loop and conduit to extend the length of the free flap for venous drainage.

**Results:**

The most common reconstructions were anterolateral thigh flaps (15 cases), followed by vastus lateralis myocutaneous (3 cases) and radial forearm (2 cases) flaps. The common recipient vessels were superior thyroid artery, superficial temporal artery and external jugular vein. The free flap loss rate was 7.7% with vein grafts and 4.9 without vein grafts (*p* = 0.380). The free flap complication rate was 50.0% and 16.8% in patients with and without vein grafts, respectively (*p* < 0.001). Radiation therapy, chemotherapy, prior neck dissection, and prior free flap transfer were more common in the vein graft group (all *p* < 0.001). The hospital stay was significantly longer for the vein graft group than for the non–vein graft group (29.5 vs. 19.0 days; *p* = 0.001).

**Conclusion:**

Overall free flap survival rates of 92.3% and 95.1% in the vein and non-vein graft groups, respectively – indicating the reliability of the vein grafts in challenging head and neck reconstructions, particularly in salvage cases and patients with multiple reconstructions.

**Level of evidence:**

Level 3.

## Highlights

• Vein grafting is a reliable option for challenging head and neck reconstruction.
• Vein grafting is a reliable option in free flap salvage cases.
• High free flap survival rate shows vein graft reliability.
• An increased postoperative complication in free flap with vein grafting is noted.

## Introduction

From functional and cosmetic perspectives, microvascular techniques are associated with substantial head and neck reconstruction defects. Currently, free tissue transfer is widely accepted as the method of choice for reconstruction in complex cases. Selecting an adequate recipient vessel is critical for efficient free flap reconstruction. However, due to anatomical variations or previous neck dissection, external irradiation, chemotherapy, or some combination of these, local recipient vessels may be unavailable. In addition, free tissue reconstruction can be complicated by a lack of healthy recipient vessels. In such circumstances, vein grafting is required to elongate free flap pedicles to connect them to appropriate recipient vessels.

Since Crowell et al.^1^ first described microvascular autografting techniques in the late 1960s, conflicts related to the reliability of free tissue transfer using vein grafts have arisen. Some reports have demonstrated a high incidence of thrombotic events and free flap loss with vein graft use.^2^, ^3^, ^4^, ^5^, ^6^, ^7^, ^8^, ^9^ However, others have not indicated an increased free flap failure risk compared with standard free flap reconstruction.^10^, ^11^, ^12^, ^13^, ^14^, ^15^, ^16^, ^17^, ^18^ Because of controversy regarding the use of interposition vein grafts for free tissue reconstruction, this paper reports vein graft indications, techniques, safety, and outcomes for head and neck microvascular surgery conducted between January 2013 and June 2018.

## Materials and methods

From January 2013 to June 2018, a single academic medical center performed 756 free flap reconstructions. We collected data, including patient demographics, surgical history, prior irradiation and chemotherapy, flap type, diagnosis, specific vein graft use, surgical outcomes, flap compromise, morbidity in flap donor site, and length of hospital stay, were collected for analysis in this retrospective case series. We identified patients who underwent interposition vascular grafting concurrent with free tissue transfer and evaluated their data.

After we conducted ablative surgery, debridement, or scar contracture release, we performed microvascular reconstruction for all vascular anastomoses immediately in a standardized manner without a coupling device. We performed all vascular anastomoses under an operating microscope, and 9–0 nylon was used. We performed conventional end-to-end microanastomosis with the interrupted suture technique. In each patient, according to the area and tissue composition of the defect after ablation, we selected a suitable donor site for free flap harvesting. We determined vein graft indications when recipient vessels in the neck were evaluated. We prepared a single interposition graft with consideration for the predicted disparity between the recipient and donor artery or vein. However, we prepared a long vein graft for bridging the gap between both an artery and a vein to the designated vessels when suitable recipient vessels on the ipsilateral neck were depleted. The interposition vein graft source was dependent on the availability of vessels at the donor site of the free flap. The Great Saphenous Vein (GSV) was the most frequently used vein graft. Postoperative care in an intensive care unit was arranged for each patient; hourly flap monitoring was provided by nursing staff for the first 48 h. Clinical monitoring included monitoring of flap skin color, surface temperature, capillary refill time, and acoustic cutaneous Doppler to detect intravascular arterial blood flow. When their condition was stable, the patients were discharged and asked to attend regular follow-ups provided by the relevant surgical teams. Morbidity at the donor site including wound dehiscence, poor wound healing, hematoma, seroma, and skin graft loss postoperatively requiring wound treatment or surgical intervention.

We used frequencies and percentages to summarize the clinical data and outcomes. The Chi-Squared, Fisher’s exact, or Mann–Whitney *U* test was used to assess associations between baseline parameters and vein grafting outcomes. A *p*-value of <0.05 was considered significant. Within-patient correlations were assessed using univariate and multivariable generalized estimating equation models to measure the effect of vein grafting on take-back for vascular exploration, partial skin necrosis of the flap, and flap loss. We adjusted odd ratios by using a multiple logistic regression model adjusting for potential confounding factors. We employed a backward model selection approach to select the predictive factors. Variables with a value of *p* ≥ 0.05 were removed from the multiple logistic regression model. All tests were two-sided. We analyzed all data by using the Statistical Package for the Social Sciences, version 22.0 (2013 release: IBM Corp., Armonk, NY, USA). Again, a two-tailed *p*-value of <0.05 denoted statistical significance. This study was conducted with the approval of the institutional review board (approval number CG19139B).

## Results

In this study, vein grafts were required in 26 patients (23 men and 3 women) with head and neck defects, accounting for 3.44% of all microvascular reconstructions (n = 756). Oral cavity reconstruction after surgical ablation of squamous cell carcinoma was the most common indication for free flap transfer (69.5%).

Interposition vein grafting methods were used in four clinical scenarios. The most common scenario was vein graft use in a planned manner because appropriate recipient vessels were unavailable in patients with tumor recurrence (n = 12, 46.2%). In the second scenario, 10 patients (38.5%) were given interposition vein grafts for flap salvage due to postoperative thrombotic events. In this scenario, first, second, and third microsurgical reconstructions were performed in four, three, and three patients, respectively. In the third scenario, three patients received flap reconstruction for the repair of a previously applied flap (n = 3, 11.5%) due to complications such as osteoradionecrosis, scar contracture, and iatrogenic infection. Finally, the fourth scenario was the application of a vein graft in an unplanned intraoperative approach (n = 1, 3.8%).

In the vein grafting series, 15 flaps (57.7%) were anterior lateral thigh flaps; 3 (11.5%) were Vastus Lateralis Myocuataneous (VLMC) flaps, and two each were radial forearm flaps (7.7%), posterior medial thigh flaps (7.7%), and fibula osteocutaneous flaps (7.7%). The most common vein graft source was the GSV (n = 22, 84.6%). We measured vein graft length after vascular anastomosis, and the average vessel graft length required was 16.1 ± 6.7 cm (range: 4–30 cm).

**Figure 1:**
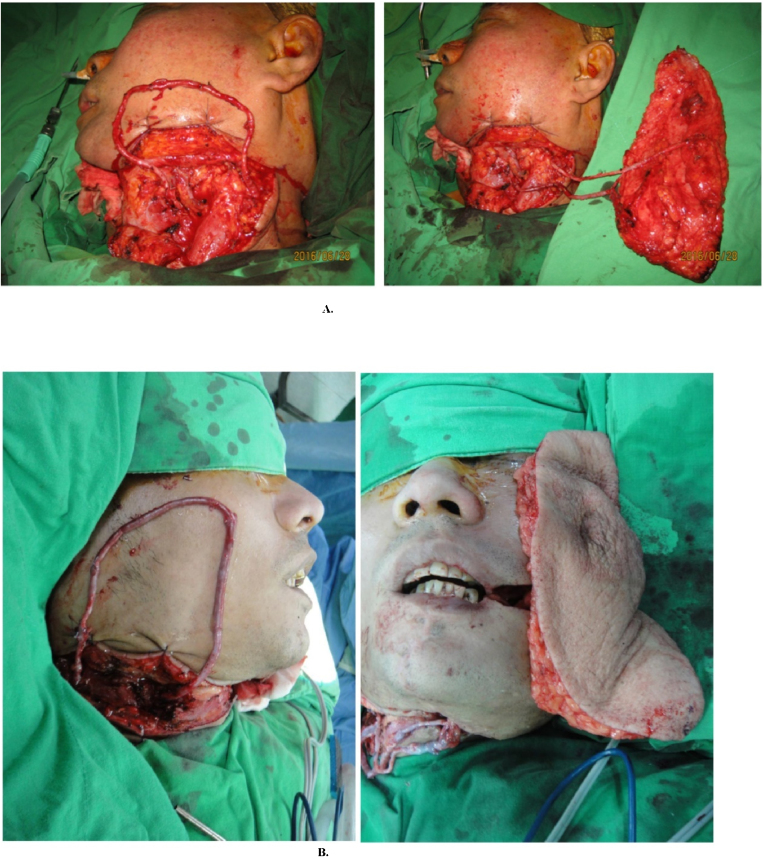
A, (Left) Creation of an arteriovenous (A–V) loop by using a 20-cm-long vein graft on the left neck. (Right) After physiological distension of the graft, the vein graft was divided at the midpoint to become two interposition vein grafts with adequate length to anastomose the pedicle of the free Anterior Lateral Thigh (ALT) flap. The flap can reach the complex defect on the right side of the face. B, (Left) Creation of an A–V loop using a 22-cm-long vein graft on the right neck. (Right) After physiological distension of the graft, the vein graft was divided at the appropriate point to anastomose the pedicle of the free ALT flap. The flap can reach the complex defect on the left side of the face.

We applied interposition vein grafts in two manners. First, we employed such grafts when suitable recipient vessels were completely depleted in the ipsilateral neck of patients with complicated conditions. The most common technique was the creation of an arteriovenous (A–V) loop; this was performed in 14 patients (53.8%). A selected recipient artery and vein at the remote site of the neck was connected using a long vein graft. The anastomoses were released to allow vein graft perfusion, creating a temporary A–V loop. The A–V loop was physiologically distended through arterial inflow. After an observation period, we divided the vein graft at the midpoint such that it became two interposition vein grafts, connecting it to the artery and vein of the flap. With this approach, the flap can reach the contralateral side of the recipient vessels (Fig. 1A and B). Second, in the 12 other patients (46.2%), we harvested vein grafts as conduits to extend the length of the free flap for venous drainage. When a pedicle had more than one vein, we harvested a vein graft with a branch for more than one venous anastomosis or else joined the vein of a pedicle with a strong venous flow.

In patients who received interposition vein grafts, the common recipient arteries were the superior thyroid artery (n = 5, 33.3%) and the superficial temporal artery (n = 5, 33.3%). The most common recipient vein was the external jugular vein (n = 19, 73.1%); we used contralateral vessels as recipient vessels in 12 patients (46.2%). In the two procedures with flap failure, one patient underwent subsequent free flap surgery, and the other was treated using pedicled pectoralis major myocutaneous flap. Complications included wound dehiscence 7 (26.9%), partial flap necrosis 3 (11.5%), wound infection 1 (3.8%), hematoma 1 (3.8%) and orocutaneous fistula 1 (3.8%). We performed skin graft operations for four patients with minor complications, and the remaining patients received wound care. The total complication rate (total flap failure excluded) was 50%.

Compared with patients in the non–vein graft group, the patients in the vein graft group were associated with a higher incidence of adjuvant therapy for their original cancers. The proportions of patients who received prior radiation therapy (84.6% vs. 40.7%; *p* = 0.001) or chemotherapy (80.8% vs. 40.0%; *p* < 0.001) were higher in the vein graft group than in the group not given vein grafts. More patients in the vein graft group had prior neck dissection and prior free flap transfer (all *p* < 0.001) (Table 1).

**Table 1:** Patient characteristics.

Characteristic	Vein graft (%)	No vein graft (%)	*p*-Value
Nº of flaps	26	730	
Demographics			
Mean age (years)	50.2 ± 8.7	54.1 ± 9.8	0.064^a^
Male sex	23 (88.5)	687 (94.1)	0.206^b^
Smoking	21 (80.8)	607 (83.2)	0.789^b^
Betel nut	19 (73.1)	559 (76.6)	0.859^c^
Alcohol use	19 (73.1)	499 (68.4)	0.768^c^
HTN	7 (26.9)	204 (27.9)	1.000^c^
DM	4 (15.4)	112 (15.3)	1.000^b^
Liver cirrhosis	0 (0)	13 (1.8)	1.000^b^
Renal failure	0 (0)	4 (0.5)	1.000^b^
Risk factors			
Prior radiation therapy	22 (84.6)	297 (40.7)	<0.001^c^
Prior chemotherapy	21 (80.8)	292 (40.0)	<0.001^c^
Prior neck dissection	22 (84.6)	329 (45.1)	<0.001^c^
Prior free flap reconstruction	21 (80.8)	254 (34.8)	<0.001^c^

DM, diabetes mellitus; HTN, hypertension.

a Mann–Whitney *U* test.

b Fisher’s exact test.

c Chi-squared test.

The free flap loss rates of the two groups did not significantly differ (*p* = 0.380; 7.7% with vein grafting and 4.9% without vein grafting). The free flap postoperative complication rate (e.g., wound infection, wound dehiscence, hematoma, partial skin necrosis of the flap, or orocutaneous fistula) was significantly higher in the vein graft group compared with the non–vein graft group (50.0% vs. 16.8%; *p* < 0.001). The take-back rate for vascular exploration was not significantly different between the two groups (*p* = 1.000; 7.7% with vein grafting and 9.2% without vein grafting). Flap donor site morbidity of the two groups did not significantly differ (*p* = 0.056; 11.5% with vein grafting and 3.2% without vein grafting). The hospital stay was significantly longer for the vein graft group than for the non–vein graft group (29.5 vs. 19.0 days; *p* = 0.001) (Table 2).

**Table 2:** Surgical outcomes.

Surgical outcome	Vein graft (%)	No vein graft (%)	Pc
Flap loss	2 (7.7)	36 (4.9)	0.380^a^
Post-operative complication	13 (50)	123 (16.8)	<0.001^a^
Take back for vascular exploration	2 (7.7)	67 (9.2)	1.000^a^
Flap donor site morbidity	3 (11.5)	23 (3.2)	0.056^a^
Length of hospital stay (days)	29.5 (11.0–126.0)	19.0 (8.0–123.0)	0.001^b^

a Fisher’s exact test.

b Mann–Whitney *U* test.

A univariate general estimating equation model was used to identify variables affecting take-back for vascular exploration and flap loss (Table 3, Table 4). None of vein grafting, prior radiation, prior chemotherapy, prior free flap reconstruction, or demographic characteristics were associated with significant risk of take-back for vascular exploration and flap loss.

**Table 3:** Univariate and multivariable generalized estimating equation model of take-back for vascular exploration.

		Univariate analysis		
		OR	(95% CI)	*p*-Value
Age > 60 yr	16/204 (7.8)	0.80	(0.45–1.44)	0.457
Sex	62/708 (8.8)	0.56	(0.24–1.31)	0.180
Vein grafting	2/26 (7.7)	0.82	(0.19–3.57)	0.796
Prior radiation therapy	34/320 (10.6)	1.36	(0.83–2.24)	0.222
Prior chemotherapy	30/309 (9.7)	1.12	(0.68–1.85)	0.644
Prior free flap reconstruction	43/416 (10.3)	1.39	(0.84–2.32)	0.203
HTN	18/222 (8.1)	0.84	(0.48–1.47)	0.531
DM	13/121 (10.7)	1.24	(0.66–2.35)	0.501
Liver cirrhosis	1/12 (8.3)	0.90	(0.11–7.11)	0.923
Renal failure	0/3 (0)			
Contralateral recipient vessels	10/106 (9.4)	1.04	(0.52–2.11)	0.906

DM, diabetes mellitus; HTN, hypertension.

**Table 4:** Univariate and multivariable generalized estimating equation model of flap loss.

		Univariate analysis		
		OR	(95% CI)	*p*-Value
Age > 60 yr	6/204 (2.9)	0.49	(0.20–1.20)	0.118
Sex	34/708 (4.8)	0.55	(0.19–1.63)	0.285
Vein grafting	2/26 (7.7)	1.61	(0.37–7.06)	0.530
Prior radiation therapy	22/320 (6.9)	1.94	(1.00–3.75)	0.050
Prior chemotherapy	19/309 (6.1)	1.48	(0.77–2.84)	0.243
Prior free flap reconstruction	26/416 (6.3)	1.82	(0.91–3.67)	0.093
HTN	7/222 (3.2)	0.53	(0.23–1.22)	0.134
DM	5/121 (4.1)	0.79	(0.30–2.06)	0.624
Liver cirrhosis	0/12 (0)			
Renal failure	0/3 (0)			
Contralateral recipient vessels	5/106 (4.7)	0.93	(0.35–2.43)	0.875

DM, diabetes mellitus; HTN, hypertension.

A univariate general estimating equation model was used to identify variables affecting partial skin necrosis of the flap (Table 5). Only prior radiation therapy and prior free flap reconstruction were associated with a significantly high risk of partial skin necrosis of the flap. Age over 60-years was associated with a significantly low risk of partial skin necrosis of the flap. In the multivariable general estimating equation model, the adjusted odds ratio for partial skin necrosis of the flap with age over 60 years was 0.46 (95% CI, 0.21–1.00; *p* = 0.049).

**Table 5:** Univariate and multivariable generalized estimating equation model of partial skin necrosis of the flap.

		Univariate analysis			Multivariable model		
		OR	(95% CI)	*p*-Value	OR	(95% CI)	*p*-Value
Age > 60 yr	8/204 (3.9)	0.45	(0.21–0.97)	0.041^a^	0.46	(0.21–1.00)	0.049^a^
Sex	50/708 (7.1)	0.84	(0.29–2.42)	0.741			
Vein grafting	4/26 (15.4)	2.47	(0.82–7.45)	0.108			
Prior radiation therapy	31/320 (9.7)	1.93	(1.10–3.37)	0.022^a^	1.35	(0.68–2.66)	0.393
Prior chemotherapy	28/309 (9.1)	1.61	(0.93–2.81)	0.091			
Prior free flap reconstruction	39/416 (9.4)	2.24	(1.21–4.14)	0.010^a^	1.85	(0.88–3.89)	0.107
HTN	12/222 (5.4)	0.67	(0.35–1.30)	0.234			
DM	7/121 (5.8)	0.77	(0.34–1.74)	0.528			
Liver cirrhosis	0/12 (0)						
Renal failure	1/3 (33.3)	6.60	(0.59–74.02)	0.126			
Contralateral recipient vessels	8/106 (7.5)	1.07	(0.49–2.34)	0.862			

DM, diabetes mellitus; HTN, hypertension.

Logistic regression.

a *p* < 0.05.

## Discussion

Theoretically, interposition vein grafting offers benefits of increased vascular pedicle length to overcome problems of tension and gaps between free flaps and recipient vessels. However, vein grafting techniques in microsurgery are far more difficult than primary anastomosis of free flap vessels because the vessel caliber of the graft may vary, and more vascular anastomoses are required.

Miller et al.^4^ and Maricevich et al.^8^ support our findings: they revealed that vascular grafting is typically used in the context of tumor recurrence, infection, flap death, other flap complications, or in patients with a history of radiotherapy. In these situations, interposition vein grafting is required due to a lack of adequate recipient vessels in a neck that has been previously operated on and irradiated. Such a scenario may decrease the quality of available vessels and cause fibrosis in the surrounding soft tissue. Studies have demonstrated a higher risk of flap failure and vascular thrombosis when microsurgery reconstruction is performed using vein grafts in patients with such conditions.^2^, ^3^, ^4^, ^5^, ^6^, ^7^, ^8^, ^9^ Therefore, microsurgeons may hesitate to use interposition vessel grafts.

Furr et al.^10^ stated that in published reports, researchers have been inconsistent in their flap failure risk estimation when interposition vascular grafting is necessary. In addition, past years studies have reported that vessel grafts can be reliable and have a low flap failure risk.^11^, ^12^, ^13^, ^14^, ^15^, ^16^, ^18^ In our study, the free flap loss rate in vein-grafted free flaps compared with non-vein grafted free flaps was not significantly higher (7.7% vs. 4.9%, respectively). In univariate analyses, the adjusted odds ratio was 1.61 (95% CI, 0.37–7.06; *p* = 0.530) for free flap loss in vein-grafted flaps. In 2018, Maricevich et al.^8^ published a study involved 241 patients undergoing free flap reconstruction with vein grafts and reported a relatively low flap loss rate (6.6%). The flap failure rate in interposition vascular grafting studies is remarkably close to that of our study (7.7%). Table 6 presents a comparison of studies involving head and neck reconstruction with free flaps and interposition vessel grafts over the past 25 years.^6^, ^8^, ^10^, ^19^^,^^20^ The flap failure rates in free tissue reconstruction with vascular grafts exhibit a gradually declining trend. This may be related to improved microsurgical techniques, refinement of vessel graft handling approaches, and progress in flap monitoring.

**Table 6:** Retrospective studies involving head and neck free flap reconstruction using interposition vascular grafts.

References	Total flaps	With vessel grafts	
		Nº of flaps (%)	Flap failure (%)
Schusterman et al.^6^	308	17 (5.5)	5 (29)
Jones et al.^16^	305	20 (6.5)	7 (35)
Podrecca et al.^17^	346	8 (2.3)	2 (25)
Furr et al.^7^	1143	20 (1.7)	1 (5.0)
Maricevich et al.^15^	3240	241 (7.4)	16 (6.6)
Lai et al.	756	26 (3.4)	2 (7.7)

Schanzer et al.^21^ suggested that technical factors, including conduit quality and handling, site choice, and runoff bed resistance, are the key determinants of perioperative success related to vein graft surgery. In our study, we considered that the cornerstone of successful free tissue transfer with interposition vein grafting is the selection of appropriate and healthy recipient arteries and veins. Because the compromised flaps of 10 patients were salvaged using interposition vein grafts, we suggest that using vascular grafts can reduce the undue tension caused by vessel anastomosis and avoid the use of recipient vessels in irradiated or fibrotic zones.

Vein graft handling and anastomosis techniques are other crucial factors affecting flap survival rate. In our study, a temporary A–V loop was created in 14 patients. Such a loop allows physiological distension of the vein graft, ensures no kinks or twists are present, and can be used to seal leaks of graft during observation periods.^22^, ^23^, ^24^, ^25^ Delicate adventitia splitting of vascular grafts is another essential technique for improving treatment outcomes. In total, two flaps were lost in the A–V loop group: one was related to hematoma and pedicle twisting, whereas the other was related to arterial thrombosis. Although using the A–V loop technique led to the loss of two flaps, the failure rate of free flap reconstruction was considerably improved compared with those in previous studies.^6^, ^19^, ^20^

Furr et al.^10^ suggested that patients undergoing interposition vein grafting are already disadvantaged by unhealthy recipient vessels and fibrotic soft tissues. Using univariate and multivariable analysis, Maricevich et al.^8^ concluded that vein grafting and prior chemotherapy are crucial factors affecting flap loss. This finding was similar to one in our study; two patients with flap failure had previously undergone chemotherapy. Recently, the total flap failure rate of free flap reconstruction with interposition vascular grafting in the head and neck has improved considerably. However, in the present study, we noted a higher complication rate (50%) in the vein graft group than in the group without vein grafts. This phenomenon was evident in Maricevich et al.,^8^ who reported a flap compromise rate of 14.5% in a vein grafting group. They defined flap compromise as free flap vascular perfusion resulting in an unplanned return to the operating room for attempted flap salvage. However, despite an increased revision rate, the overall flap survival rate was unaffected. Overall free flap survival rates of 92.3% and 95.1% in the vein and non–vein graft groups, respectively, indicates the reliability of vein grafting for challenging head and neck reconstruction, especially in salvage scenarios and in patients with multiple prior reconstructions. Careful flap monitoring and early detection of compromised flaps are essential for patients requiring interposition vein grafting with microvascular reconstruction. In addition, decisions on when to use vessel grafts should be evaluated in advance. This is consistent with the experiences of Acland and Nelson,^26^, ^27^ who have indicated that planned vein grafts are generally associated with favorable graft survival. Although the difference in flap donor site morbidity between the groups in our study did not reach statistical significance, with the additional skin incision for harvesting vein graft and longer operation time for extra micro-anastomoses in the vein graft group, a higher incidence of flap donor site morbidity can be expected when the size of a vein graft group matches that of a control group.

This study had several limitations. First, the small number of included patients means that the results may not be generalizable to other populations,^6^, ^7^, ^15^, ^16^, ^17^ even though this study was the second-largest case series among recent publications (Table 6). Second, because of the small number of patients, exploring whether vessel graft length affects the incidence of thromboembolism was not possible. Determining whether flap type affects operation success and complication rates was also impossible. Third, this was a retrospective study, and such studies have inherent limitations related to the level of evidence.

## Conclusion

Interposition vein grafts can be used to salvage compromised free flaps; they are most often used in patients who have undergone multiple microvascular flap surgeries of the head and neck. Although an increased postoperative complication risk was noted compared with standardized surgical techniques, vein grafts have a high success rate, even in difficult cases. The selections of healthy recipient arteries and veins as well as meticulous flap monitoring are the key determinants of successful microvascular surgery using interposition vein grafts. It is a reliable option for challenging head and neck reconstruction and a valuable alternative for salvage cases.

## Author contributions

Study conception and design: Chih-Sheng Lai, Ching-Hui Shen, Yi-Ting Chang, Yi-Ling Lin. Acquisition of data: Chih-Sheng Lai, Yi-Ling Lin, Yueh-Chi Tsai. Drafting of data: Chih-Sheng Lai, Chen-Te Lu, Yueh-Chi Tsai, Jung-Hsing Yen. Critical revision: Chih-Sheng Lai, Yueh-Chi Tsai, Yi-Ling Lin, I-Chen Chen.

## Funding

This research did not receive any specific grant from funding agencies in the public, commercial, or not-for-profit sectors.

## Conflicts of interest

The authors declare no conflicts of interest.
